# A prognostic score model to determine which breast cancer patients with 1–3 positive lymph nodes after modified radical mastectomy should receive radiotherapy

**DOI:** 10.18632/oncotarget.21531

**Published:** 2017-10-05

**Authors:** Dawei Chen, Haiyong Wang, Xinyu Song, Fang Shi, Li Kong, Jinming Yu

**Affiliations:** ^1^ Department of Radiation Oncology, Shandong Cancer Hospital affiliated with Shandong University, Shandong Academy of Medical Sciences, Shandong, China; ^2^ Department of Internal Medicine-Oncology, Shandong Cancer Hospital affiliated with Shandong University, Shandong Academy of Medical Sciences, Shandong, China; ^3^ School of Medicine and Life Sciences, University of Jinan-Shandong Academy of Medical Sciences, Shandong, China

**Keywords:** breast cancer, postmastectomy radiotherapy, SEER, population-based study

## Abstract

There is no consensus on the indication for postmastectomy radiotherapy (PMRT) in breast cancer patients with one to three positive lymph nodes. To identify patients for whom PMRT may be indicated, we used a prognostic score model with the SEER database to retrospectively analyze 8049 patients with one to three positive lymph nodes who underwent mastectomy with or without PMRT between 2010 and 2013. Kaplan-Meier analysis showed that PMRT patients had better overall survival (OS) than no-PMRT patients (*P* < 0.001); however, there was no difference in cancer-specific survival (CSS) (*P =* 0.530). Multivariate analysis with Cox regression showed that grade (*P* < 0.001), tumor size (*P* < 0.001), and progesterone receptor status (*P* < 0.001) were independent prognostic factors for OS. To diminish bias, we used 1:1 propensity score matching analysis and prognosis score model, which revealed that PMRT patients had better OS and CSS than no-PMRT patients (*P* < 0.001). In a concrete subgroup analysis of PMRT patients, significant improvements in OS were observed in patients scoring 0, 1, or 2. PMRT patients scoring 2 also had improved CSS. The magnitude of the OS and CSS difference with PMRT correlated with the prognostic score (*P* < 0.001). These results suggest PMRT in breast cancer patients with one to three positive lymph nodes should be based on patient factors, tumor biology, and prognostic score.

## INTRODUCTION

The incidence of breast cancer and its related deaths has increased. Clinical studies have shown that postmastectomy radiotherapy (PMRT) can improve the regional control rate and prolong overall survival (OS) when combined with systematic treatments [1–3]. PMRT is currently recommended as a standard treatment for breast cancer patients with four or more positive lymph nodes. For patients with one to three positive lymph nodes, the 10-year regional recurrence rate ranges from 12% to 27%, and there is no consensus on the use of PMRT in these patients [4–7].

Studies have attempted to determine the appropriate subgroups to receive PMRT [2, 7–15]. Several factors, including age, tumor size, grade, and surgical margin status, have been identified as correlative factors that increase the risk of regional recurrence after mastectomy, suggesting that patients at high risk after mastectomy may be suitable for PMRT.

In this study, we retrospectively analyzed 8,049 patients with one to three positive lymph nodes undergoing mastectomy with or without PMRT to identify the subgroups who benefit from PMRT.

## RESULTS

### Patient demographics

A total of 8,049 female breast cancer patients were reported in the SEER database from 2010 to 2013. The clinical characteristics and pathologic features of all patients are listed in Table 1. Most patients were diagnosed at age > 40 years (85.1% in PMRT group; 92.7% in no PMRT group). Grade 3 tumors were diagnosed in 49.3% of patients who received PMRT and 42.3% of patients who did not receive PMRT. More patients who received PMRT had larger tumors (≥ 5 cm) than patients who did not receive PMRT (25.9% vs. 10.6%, respectively). In patients with PMRT versus no PMRT, rates of ER-, PR-, and HER2-positive tumors were 78.2% vs. 81.7%, 66.7% vs. 70.1%, and 21.1% vs. 19.5%, respectively. In both groups, more patients had one positive lymph node (42.2% with PMRT and 57.6% with no PMRT). In the PMRT and no PMRT groups, 59.1% and 52.9% of patients were married, respectively.

**Table 1:** Characteristics of breast cancer patients with 1–3 positive lymph nodes from SEER database

Variable	No. of patients with PMRT (%)	No. of patients without PMRT (%)	*P*
**Total**	3,372	4,677	
**Age**			< 0.001
< 40	501 (14.9)	341 (7.3)	
40–59	1,801 (53.4)	2,071 (44.3)	
≥ 60	1,070 (31.7)	2,265 (48.4)	
**Grade**			< 0.001
1 and 2	1,710 (50.7)	2,700 (57.8)	
3	1,662 (49.3)	1,977 (42.3)	
**Laterality**			0.138
Right	1,688 (50.0)	2,263 (48.4)	
Left	1,684 (49.9)	2,414(51.6)	
**Tumor size**			< 0.001
< 5 cm	2,499 (74.4)	4,180 (89.3)	
≥ 5 cm	873 (25.9)	497 (10.6)	
**ER**			< 0.001
Positive	2,636 (78.2)	3,821 (81.7)	
Negative	736 (21.8)	856 (18.3)	
**PR**			0.001
Positive	2,250 (66.7)	3,278 (70.1)	
Negative	1,122 (33.3)	1,399 (29.9)	
**HER2**			0.071
Positive	712 (21.1)	911 (19.5)	
Negative	2,660 (78.9)	3,766 (80.5)	
**Positive nodes**			< 0.001
1	1,424 (42.2)	2,692 (57.6)	
2	1,118 (33.2)	1,335 (28.5)	
3	830 (24.6)	650 (13.9)	
**Nodes examined**			0.658
< 10	1,048 (31.1)	1,439 (30.8)	
10–20	1,842 (55.6)	2,597 (55.5)	
> 20	482 (14.3)	641 (13.7)	
**Married**			< 0.001
Yes	1,994 (59.1)	2,473 (52.9)	
No	1,378 (40.9)	2,204 (47.1)	

### Survival outcomes of patients

**Figure 1:**
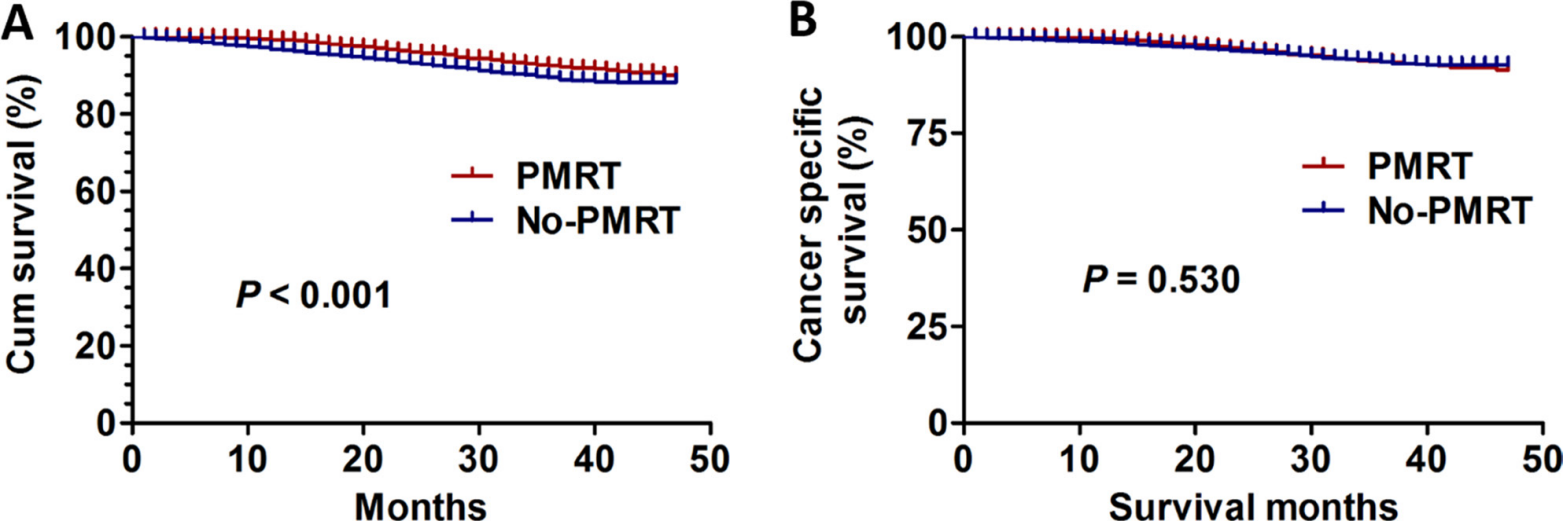
The survival curves in breast cancer patients with PMRT and no PMRT between 2010 and 2013 **(A)** OS curves (χ2 = 22.70, *P* <0.001). **(B)** CSS curves (χ2 = 0.395, *P* = 0.530).

Breast cancer patients with PMRT had better OS compared with patients with no PMRT (χ2 = 22.70, *P* <0.001) (Figure 1A). However, difference in CSS between the two groups was not significant (χ2 = 0.395, *P* = 0.530) (Figure 1B).

### Prognostic risk factors analysis for patients with PMRT

Univariate and multivariate Cox proportional hazards regression analysis was performed to analyze the prognoses of patients who received PMRT. Results showed that age, grade, laterality, tumor size, and ER and PR status were significant risk factors for OS using univariate analysis (all *P* > 0.05) (Table 2). Multivariate analysis with Cox regression was then performed and found that only grade (hazard ratio [HR] 2.998; 95% confidence interval [CI] 1.780–5.050; *P* < 0.001), tumor size (HR 2.423; 95% CI 1.680–3.495; *P* < 0.001), and PR status (HR 2.848; 95% CI 1.688–4.804; *P* < 0.001) were independent prognostic factors for OS (Table 2).

**Table 2:** Univariate and multivariate Cox proportional hazards regression analysis of OS in patients who underwent PMRT

Variable	Univariate analysis		Multivariate analysis	
	Log-rank χ2 test	*P*	HR (95% CI)	*P*
**Age**	6.192	0.045		0.204
< 40			reference	
40–59			0.662 (0.409–1.072)	0.094
≥ 60			0.847 (0.509–1.411)	0.525
**Grade**	57.976	< 0.001		< 0.001
1 and 2			reference	
3			2.998 (1.780–5.050)	< 0.001
**Laterality**	0.036	0.850		
Right				
Left				
**Tumor size**	31.290	< 0.001		< 0.001
< 5 cm			reference	
≥ 5 cm			2.423 (1.680–3.495)	< 0.001
**ER**	76.849	< 0.001		0.247
Positive			reference	
Negative			1.322 (0.824–2.122)	0.247
**PR**	84.628	< 0.001		< 0.001
Positive			reference	
Negative			2.848 (1.688–4.804)	< 0.001
**HER2**	3.477	0.062		
Positive				
Negative				
**Positive nodes**	4.600	0.100		
1				
2				
3				
**Nodes examined**	0.953	0.813		
< 10				
10–20				
20–30				
≥ 30				
**Married**	**1.634**	0.201		
Yes				
No				

### Risk prediction model

**Figure 2:**
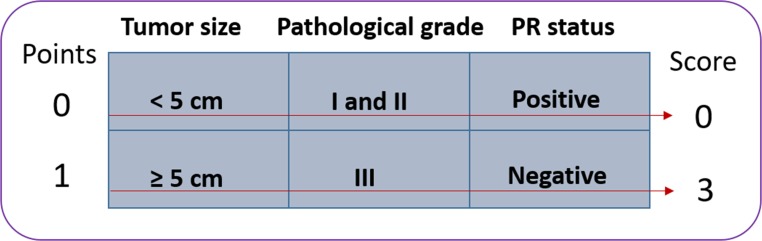
Prognostic score model for patients with one to three positive lymph nodes after modified radical mastectomy

Our study indicates that grade, tumor size, and PR status are independent prognostic factors for OS. Thus, we created a prognostic score model based on these confirmed prognostic factors for patients with PMRT, with the total number of risk factors defined as the prognostic score. Therefore, patients with a prognostic score of 0 have no risk factors, whereas patients with a prognostic score of 1, 2, or 3 have one, two, or three risk factors, respectively (Figure 2).

### Propensity score matching analysis

As shown in Table 1, the baseline clinical characteristics were significantly different between patients who received PMRT and those who did not receive PMRT. To eliminate the influence of this difference on survival, propensity score matching was conducted to reevaluate the prognosis. After performing 1:1 propensity score matching analysis, all variables were well balanced between the two groups (all *P* > 0.05) (Table 3). Interestingly, after matching, the percentages of all variables were almost exactly the same between the two groups (Table 3).

**Table 3:** Characteristics of breast cancer patients with 1–3 positive lymph nodes from SEER database after propensity score matching analysis

Variable	No. of patients with PMRT (%)	No. of patients without PMRT (%)	*P*
**Tota**l	1,800	1,800	
**Age**			> 0.05
< 40	137 (7.6)	137 (7.6)	
40–59	1,004 (55.8)	1,004 (55.8)	
≥ 60	659 (36.6)	659 (36.6)	
**Grade**			> 0.05
1 and 2	963 (53.5)	963 (53.5)	
3	837 (46.5)	837 (46.5)	
**Laterality**			> 0.05
Right	893 (49.6)	893 (49.6)	
Left	907 (50.4)	907 (50.4)	
**Tumor size**			> 0.05
< 5 cm	1,595 (88.6)	1,595 (88.6)	
≥ 5 cm	205 (11.4)	205 (11.4)	
**ER**			> 0.05
Positive	1,460 (81.1)	1,460 (81.1)	
Negative	340 (18.9)	340 (18.9)	
**PR**			> 0.05
Positive	1,311 (72.8)	1,311 (72.8)	
Negative	489 (27.2)	489 (27.2)	
**HER2**			> 0.05
Positive	277 (15.4)	277 (15.4)	
Negative	1,524 (84.7)	1,524 (84.7)	
**Positive nodes**			> 0.05
1	913 (50.7)	913 (50.7)	
2	582 (32.3)	582 (32.3)	
3	305 (16.9)	305 (16.9)	
**Nodes examined**			> 0.05
< 10	570 (31.7)	570 (31.7)	
10–20	1,004 (55.8)	1,004 (55.8)	
> 20	226 (12.6)	226 (12.6)	
**Married**			> 0.05
Yes	1,100 (61.1)	1,100 (61.1)	
No	700 (38.9)	700 (38.9)	

### Subgroup analysis for OS based on prognostic score model

**Figure 3:**
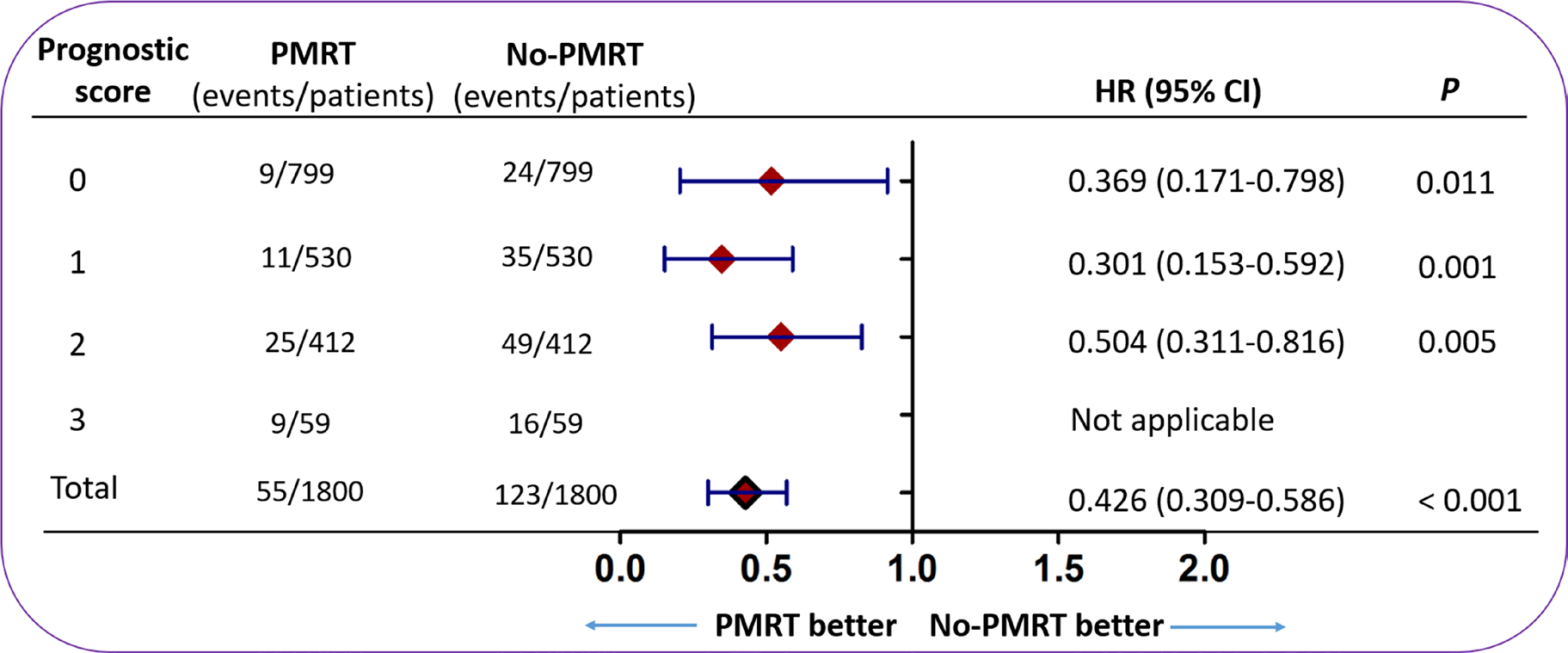
The forest plot for hazard ratio comparing OS between the PMRT group and no PMRT group according to different variables

After using propensity score matching analysis to eliminate the differences in baseline clinical characteristics, Cox proportional hazards analysis was performed to analyze the prognosis factors for OS based on the prognostic score model. Then a forest plot was applied to depict the subgroup analysis. The results showed that patients with PMRT had improved OS compared with patients without PMRT (HR 0.426; 95% CI 0.309–0.586; *P* < 0.001) (Figure 3). Importantly, for patients with a prognostic score of 3, PMRT patients did not have improved OS compared with no PMRT patients (χ2 = 1.813, *P* = 0.178) (Supplementary Figure 1). For patients with a prognostic score of 0, 1, or 2, patients who received PMRT had a greater OS benefit compared with patients who did not receive PMRT (prognostic score 0: HR 0.369; 95% CI: 0.171–0.798; *P* = 0.011; prognostic score 1: HR 0.301; 95% CI 0.153–0.592; *P* = 0.001; prognostic score 2: HR 0.504; 95% CI 0.311–0.816; *P* = 0.005) (Figure 3).

### Subgroup analysis for CSS based on prognostic score model

**Figure 4:**
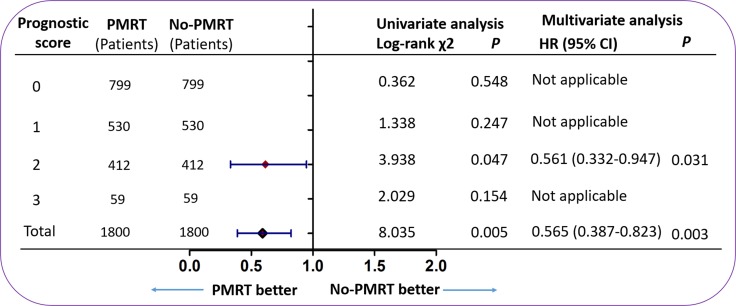
The forest plot for hazard ratio comparing CSS between the PMRT group and no PMRT group according to different variables

Cox proportional hazards analysis was done to assess the prognostic factors for CSS based on the prognostic score model. Then a forest plot was applied to depict the subgroup analysis. The results showed that patients with PMRT had improved CSS compared with patients without PMRT (HR 0.565; 95% CI 0.387–0.823; *P* = 0.003) (Figure 4). However, PMRT improved CSS only in patients with a prognostic score of 2 (HR 0.561; 95% CI 0.332–0.947; *P* = 0.031). For patients with a prognostic score of 0, 1, or 3, PMRT patients did not have improved CSS compared with no PMRT patients (prognostic score 0: *P* = 0.548; prognostic score 1: *P* = 0.247; prognostic score 3: *P* = 0.154) (Supplementary Figure 2).

## DISCUSSION

Studies have found that PMRT can reduce locoregional recurrence and improve the OS of patients with four or more positive lymph nodes. Currently, PMRT is mainly indicated in breast cancer patients with tumor diameter > 5 cm, four or more positive lymph nodes, and/or involvement of the skin or fascia of skeletal muscle. However, administration of PMRT is still controversial in patients with one to three positive lymph nodes. Therefore, clinical trials have aimed to clarify the role of PMRT in these patients. These trials include the Southwest Oncology Group 9927 trial, which was closed prematurely in 2003 because of poor recruitment [16], and another large trial (SUPREMO) that has been completed and will be reporting results soon [17].

Our study demonstrates that PMRT improved OS compared with no PMRT in breast cancer patients with one to three positive lymph nodes who received modern adjuvant systemic treatments (*P* < .001). Furthermore, using 1:1 propensity score matching analysis and a prognostic score model, we found that patients who received PMRT had better OS and CSS than patients who did not receive PMRT. These results may justify the use of PMRT in breast cancer patients with one to three positive lymph nodes.

Our results are consistent with previous studies with similar designs [18–20]. Analyzing data from the SEER registry, Buchholz et al. reported that radiation was independently associated with a survival benefit in breast cancer patients with one to three positive lymph nodes compared with mastectomy alone [18]. Kim et al. reported that in breast cancer patients with one to three positive lymph nodes who received adjuvant doxorubicin-based chemotherapy, PMRT patients had reduced locoregional recurrence and distant metastasis rates and better survival outcomes compared with no PMRT patients [19]. An earlier meta-analysis clearly demonstrated that in patients with early-stage breast cancer and positive lymph nodes, those who received PMRT had superior OS and locoregional recurrence rates (LLRs) compared with patients who did not receive PMRT [20]. However, only survival rates were presented for breast cancer patients with one to three positive lymph nodes.

Thus, evidence indicates that PMRT improves outcomes in breast cancer patients with one to three positive lymph nodes compared with no PMRT. Studies have demonstrated that PMRT, compared with no PMRT, reduces the risk of LRR in selected breast cancer patients with one to three positive nodes and several high-risk factors [7, 9–14]. However, definite risk factors have not been determined, and the criteria for identifying high-risk groups varied in these studies. In our study, tumor size, pathologic grade, and PR status were independent prognostic factors for OS. Consistent with previous studies, tumor size and pathologic grade are common high-risk factors. PR overexpression was identified as an independent risk factor for OS. However, Moo et al. reported that molecular subtype based on immunohistochemical surrogate markers was not associated with LRR in breast cancer patients with one to three positive lymph nodes treated with mastectomy [21]. Young age was commonly considered a risk factor; however, the cutoff age between old and young varied among studies [7–9, 13]. Moreover, in several studies including ours, age was not associated with OS after mastectomy [11, 12, 14, 22].

Indeterminate risk factors have raised the question of whether PMRT should be used routinely or selectively for breast cancer patients with one to three positive lymph nodes. Because the latest Oxford review demonstrated that PMRT significantly improved local control and survival rates after axillary clearance and systematic therapy in all patients with early breast cancer with positive lymph nodes [15], recent guidelines recommended routine use of PMRT in patients with node-positive breast cancer, irrespective of the number of positive lymph nodes [23, 24]. However, this meta-analysis included prospective randomized trials initiated prior to 2000, before the introduction of modern diagnostic procedures and systematic treatments. Because of this limitation, several investigators have disagreed with the routine use of PMRT based on the fact that improved diagnostic procedures and systemic therapy, as well as increased selective use of PMRT for high-risk patients, led to a lower LRR rate among patients who did not receive PMRT [13, 25]. Moo et al. reported similar LRR rates in the PMRT and no PMRT groups (3.2% vs. 4.3%, respectively) [13]. In addition, an MD Anderson Cancer Center study reported that patients treated more recently (2000–2007) who did not receive PMRT exhibited an extremely low 5-year LRR rate (2.8%) [25]. The authors argued that detailed pathologic processing and serial sectioning of the sentinel lymph node biopsy increased the selective use of PMRT and that the introduction of more effective systemic regimens, including taxanes and aromatase inhibitors, resulted in favorable locoregional outcomes in patients who did not require PMRT.

Although the low LRR rate from recent studies limited the role of PMRT for locoregional control, PMRT still seemed to improve CSS in the era of modern diagnostic and therapeutic procedures. Chang et al. reported that, although advances in diagnostic procedures and systemic treatments reduced the LRR rate among patients who did not undergo PMRT to a rate similar to that in patients who received PMRT, PMRT increased CSS significantly [22]. Our study also suggests that PMRT improves survival in breast cancer patients with one to three positive nodes.

Recently, the Medical Research Council and European Organization for Research and Treatment of Cancer initiated a prospective randomized trial, called SUPREMO, to investigate the survival benefit of PMRT in early-stage breast cancer patients with intermediate risk [16]. The results of this trial will provide guidelines for PMRT in breast cancer patients with one to three positive nodes.

Our retrospective study had several limitations, including a short follow-up time and the intrinsic defects of nonrandomized retrospective studies. Many factors, such as comorbidities, surgical margin status, adjuvant chemotherapy regimens, targeted therapies, and blood vessel invasion, were not available in the SEER database and may have influenced overall results; thus, our results should be interpreted with caution. Decisions regarding PMRT should be made based on patient factors, tumor biology, and the prognostic score. Patients with smaller tumors and fewer involved lymph nodes were more likely to receive PMRT. This bias might have seriously affected treatment outcomes. Despite our efforts to adjust for this bias via multivariate analysis, it is likely that other unknown biases influenced our results. Insufficient use of regional nodal irradiation was also a limitation of this study.

In conclusion, use of PMRT in breast cancer patients with one to three positive lymph nodes should be based on patient factors, tumor biology, and prognostic score.

## MATERIALS AND METHODS

### Patient selection

The Surveillance, Epidemiology, and End Results (SEER) Cancer Statistics Review (http://seer.cancer.gov/data/citation.html) is published annually by the Data Analysis and Interpretation Branch of the National Cancer Institute. Eighteen population-based cancer registries in the United States are included in the current SEER database [11]. The SEER*Stat software (version 8.3.2) was used to identify the appropriate patients. Using this software, we screened female breast cancer patients between 2010 and 2013. Included patients had to meet the following criteria: diagnosis confirmed microscopically, female sex with confirmed age, active follow-up, and only one primary tumor. In addition, patients had to have received modified radical mastectomy, with one to three positive lymph nodes removed. Patients with benign or borderline tumors were excluded. Patients without the following information were also excluded: age; grade; laterality; estrogen receptor (ER), progesterone receptor (PR), and human epidermal growth factor receptor 2 (HER2) status; number of positive lymph nodes; number of node examined; marital status; cause of death; and survival time.

### Ethics statement

This study was mainly based on the SEER database and was conducted in compliance with the Declaration of Helsinki. We obtained permission to access the files of SEER program research data (reference number 11304-Nov 2015). Informed consent was not required because patients were not personally identified. This study was approved by the Ethics Committee of the Shandong Cancer Hospital affiliated with Shandong University.

### Statistical analysis

For all patients, the following variables were analyzed: age; grade; laterality; ER, PR, and HER2 status; number of positive lymph nodes; number of nodes examined; and marital status. OS and cancer-specific survival (CSS) were the primary end points of this study and were extracted from the SEER database. Kaplan-Meier analyses were used to generate the survival curves, and the log-rank test was used to analyze the differences among the curves. Propensity score matching analysis was used to determine the matched patients. Cox proportional hazards analysis was used to analyze survival based on different subgroup variables, and the concrete results were presented in a forest plot. All statistical tests were two-sided, and *P* < 0.05 was considered statistically significant. SPSS 22.0 statistical software (SPSS, Chicago, IL) was used for all data analysis.

## SUPPLEMENTARY MATERIALS FIGURES


