# Supplementary material for: A prognostic score model to determine which breast cancer patients with 1–3 positive lymph nodes after modified radical mastectomy should receive radiotherapy
Source: Oncotarget. 2017 Oct 5;9(1):385–93. doi: 10.18632/oncotarget.21531 (PMC5787474; doi:10.18632/oncotarget.21531)
Supplement: Supplementary file 1 [file oncotarget-09-385-s001.pdf]

A prognostic score model to determine which breast cancer patients with 1–3 positive lymph nodes after modified radical mastectomy should receive radiotherapy

SUPPLEMENTARY MATERIALS

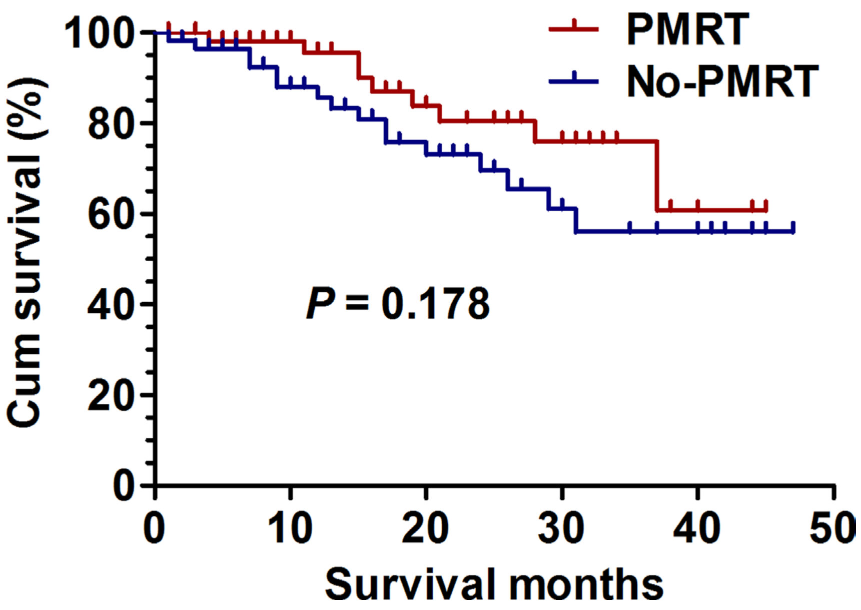

Supplementary Figure 1: OS curves for the patients with prognostic score of 3 (χ^2 = 1.813, *P* = 0.178).

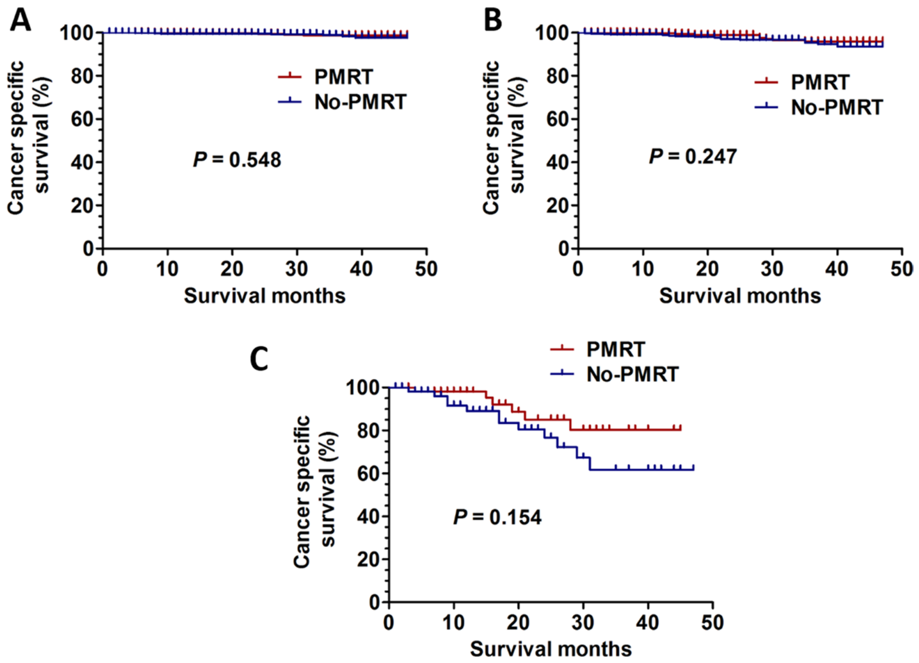

Supplementary Figure 2: CSS curves for patients with different prognostic scores. (**A**) CSS curves for patients with a prognostic score of 0 (χ^2 = 0.362, *P* = 0.548). (**B**) CSS curves for patients with a prognostic score of 1 (χ^2 = 1.338, *P* = 0.247). (**C**) CSS curves for patients with a prognostic score of 3 (χ^2 = 2.029, *P* = 0.154).
